# The ε3 and ε4 Alleles of Human APOE Differentially Affect Tau Phosphorylation in Hyperinsulinemic and Pioglitazone Treated Mice

**DOI:** 10.1371/journal.pone.0016991

**Published:** 2011-02-10

**Authors:** Alvina W. M. To, Elena M. Ribe, Tsu Tshen Chuang, Joern E. Schroeder, Simon Lovestone

**Affiliations:** 1 King's College London, Institute of Psychiatry, London, United Kingdom; 2 Stem Cell DPU, GlaxoSmithKline, Cambridge, Massachusetts, United States of America; 3 Neurosciences CEDD, GlaxoSmithKline, Harlow, United Kingdom; New York State Institute for Basic Research, United States of America

## Abstract

**Background:**

Impaired insulin signalling is increasingly thought to contribute to Alzheimer's disease (AD). The ε4 isoform of the APOE gene is the greatest genetic risk factor for sporadic, late onset AD, and is also associated with risk for type 2 diabetes mellitus (T2DM). Neuropathological studies reported the highest number of AD lesions in brain tissue of ε4 diabetic patients. However other studies assessing AD pathology amongst the diabetic population have produced conflicting reports and have failed to show an increase in AD-related pathology in diabetic brain. The thiazolidinediones (TZDs), peroxisome proliferator-activated receptor gamma agonists, are peripheral insulin sensitisers used to treat T2DM. The TZD, pioglitazone, improved memory and cognitive functions in mild to moderate AD patients. Since it is not yet clear how apoE isoforms influence the development of T2DM and its progression to AD, we investigated amyloid beta and tau pathology in APOE knockout mice, carrying human APOEε3 or ε4 transgenes after diet-induced insulin resistance with and without pioglitazone treatment.

**Methods:**

Male APOE knockout, APOEε3-transgenic and APOEε4-transgenic mice, together with background strain C57BL6 mice were kept on a high fat diet (HFD) or low fat diet (LFD) for 32 weeks, or were all fed HFD for 32 weeks and during the final 3 weeks animals were treated with pioglitazone or vehicle.

**Results:**

All HFD animals developed hyperglycaemia with elevated plasma insulin. Tau phosphorylation was reduced at 3 epitopes (Ser396, Ser202/Thr205 and Thr231) in all HFD, compared to LFD, animals independent of APOE genotype. The introduction of pioglitazone to HFD animals led to a significant reduction in tau phosphorylation at the Ser202/Thr205 epitope in APOEε3 animals only. We found no changes in APP processing however the levels of soluble amyloid beta 40 was reduced in APOE knockout animals treated with pioglitazone.

## Introduction

The concept that impaired insulin signalling influences neurodegenerative mechanisms in Alzheimer's disease (AD) and leads to impairment in cognitive processes is attracting increasing attention. This hypothesis was prompted by the clinical investigations showing the reduction of glucose utilisation in the AD brain [1]. Subsequently, experimentally induced brain insulin resistance models have shown that altered insulin signalling causes cognitive deficits and, at least in some instances influences aspects of AD pathology [2], [3]. Interestingly, reduced levels of insulin, insulin-like growth factor receptors and insulin degrading enzyme have been reported in brain tissue from AD patients [4], [5]. In addition there have been reports of low cerebrospinal fluid (CSF) to plasma insulin ratio in AD patients, which is influenced by APOE genotype [6].

The ε4 isoform of the APOE gene is the most significant genetic risk factor for AD [7]. Type 2 diabetes mellitus (T2DM), another risk factor of AD [8], [9], [10], [11], is a metabolic disorder characterised by insulin resistance, relative insulin deficiency and glucose intolerance [12]. Intriguingly, there is also some evidence of a synergistic interaction between the APOEε4 allele and T2DM. Like AD, T2DM is associated with impaired insulin signalling and cognitive functions [13]. The risk of AD is highest amongst APOEε4 carriers with T2DM [14] and neuropathological studies reported the highest number of AD lesions in brain tissue of ε4 diabetic patients [14]. However, other studies have produced conflicting findings and have failed to show an increase in AD-related pathology in diabetic brain [15], [16], [17].

Further evidence for a link between insulin signalling and AD comes from rodent models. However, as in man, these *in vivo* models are complex with some apparently contradictory evidence coming from genetic versus dietary, and from type 1 versus type 2 models. Thus, diet-induced insulin resistance accelerates β-amyloid (Aβ) pathology [18], whilst peripheral insulin injection induces tau phosphorylation [19]. Gene deletion experiments inducing insulin resistance both reduce Aβ deposition [20] and yet increase tau phosphorylation [21].

Based upon evidence from epidemiology and animal models, treatments aimed at restoring adequate levels of brain insulin have been proposed as a therapeutic approach for AD. Reger *et al*., and Craft and colleagues found that administration of insulin via peripheral [22], [23] and intranasal [24], [25] routes improved cognition and these effects were more prominent in non-APOEε4 carriers [22], [24]. Improved cognitive performance has also been demonstrated in animals treated with intranasal insulin [26], which also improved diabetic brain neuropathy [27].

The thiazolidinediones (TZDs), peroxisome proliferator-activated receptor gamma (PPARγ agonists such as rosiglitazone and pioglitazone, are widely used clinical treatments for T2DM. TZDs are insulin sensitising drugs that reduce peripheral insulin resistance and blood glucose levels [28]. In two early clinical trials, rosiglitazone improved memory and cognition in patients with mild to moderate AD [29], [30] in an APOE-dependent manner [30]. More recently pioglitazone also preserved cognitive function in AD [31]. Consistent with these observations, treatment with the same insulin sensitising agents in experimental models attenuated learning and memory deficits [32], reduced the accumulation of Aβ, reduced Aβ42 levels and reduced levels of inflammatory markers [33]. PPARγ activation has also been shown to reduce the phosphorylation of tau *in vitro* [34], whilst the overexpression of PPARγ reduces the accumulation of Aβ [35], [36].

These and many other reports lend credence to the hypothesis that brain insulin resistance leads to AD pathological processes and thereby support the notion that PPARγ agonists such as the TZDs might be useful therapeutic agents. The observations in man that increased risk of AD with T2DM is APOE dependent, that insulin affects Aβ pathology in an APOE dependent manner and that in the early clinical trials with TZDs there was a stratification of effect by APOE led to the hypothesis that induced insulin resistance and subsequent treatment with TZDs in animal models would have APOE dependent effects on ‘downstream’ AD pathology, specifically tau phosphorylation. In order to explore this we have utilised an APOE ‘humanised’ mouse model lacking the mouse APOE gene with human APOEε3 or ε4 knocked in. We induced insulin resistance in these animals and subsequently treated either with or without PPARγ treatment. In the first study, our data shows diet-induced insulin resistance reduces tau phosphorylation at 3 epitopes, independent of APOE genotype. In the second of these studies, pioglitazone treatment led to a significant reduction in tau phosphorylation at the Ser202/Thr205 epitope in APOEε3 animals only, the reverse in APOEε4 mice was observed.

## Results

### APOE mice develop diet-induced type 2 diabetes mellitus-like insulin resistance

**Figure 1:**
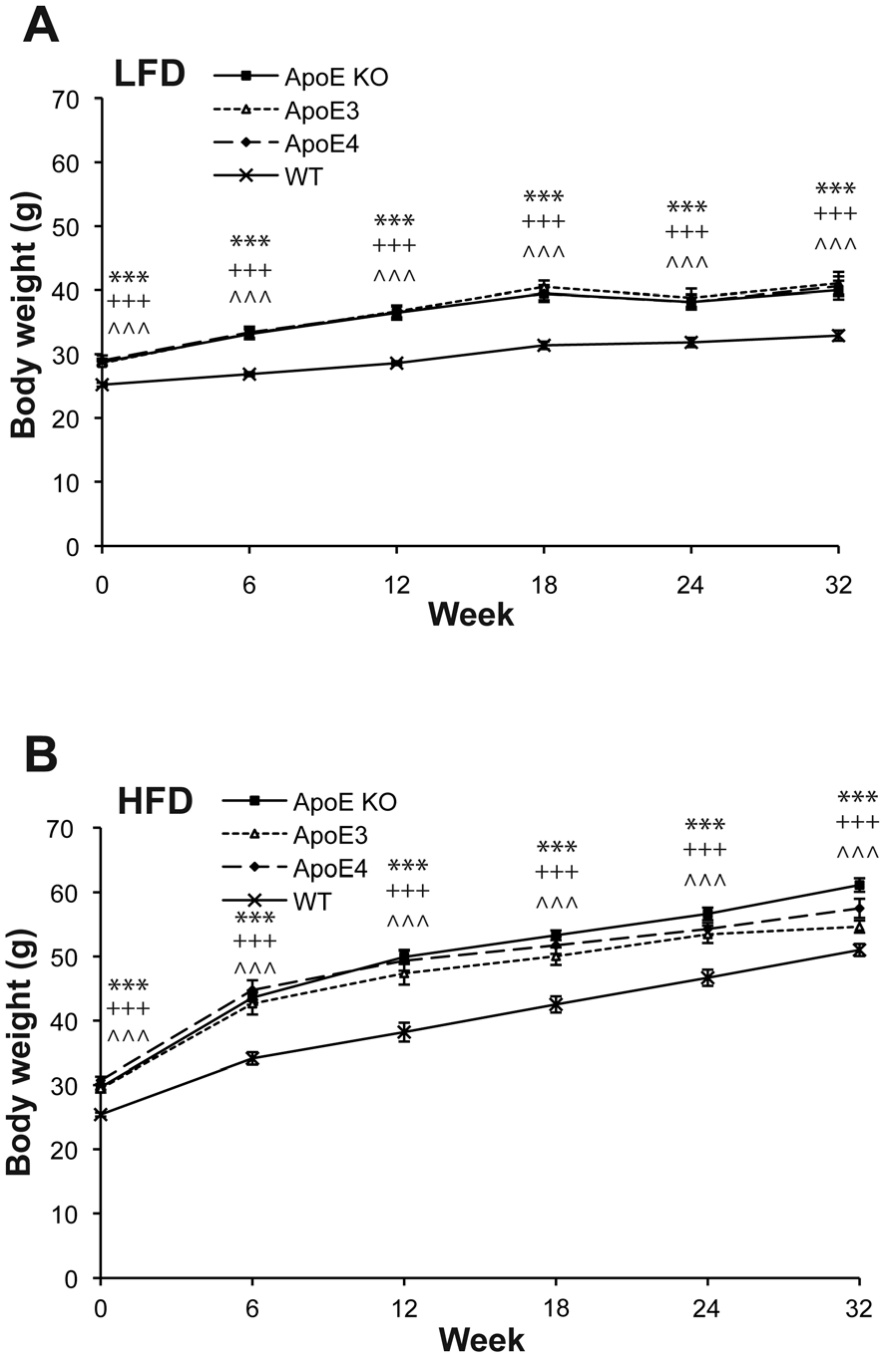
Body weight increases in HFD fed mice. Body weight of APOE KO, APOEε3, APOEε4 and WT mice over 32 weeks of A) LFD and B) HFD, starting from 3 months of age. Values are mean±SEM, n = 10-12. Body weights in A) and B) reached ***p<0.001 by t-test at all weeks indicated compared to WT mice. Significance is indicated by * APOE KO, ^+^ APOEε3, ∧ APOEε4.

**Figure 2:**
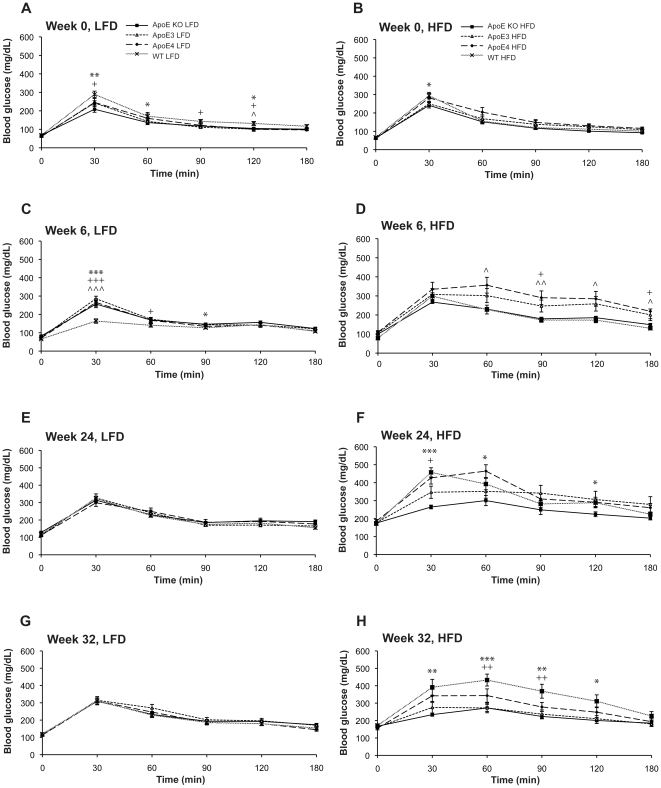
Animals develop glucose intolerance over 32 weeks of HFD. Oral glucose tolerance tests (OGTT) were conducted at baseline and then at 6 weekly intervals. Animals were fasted overnight. The next morning, animals were placed in a warming chamber prior to blood sampling and blood was taken by direct venopuncture. Mice were then given a single oral dose of glucose (3 g/kg p.o dose volume 10 ml/kg) and serial blood samples collected from the tail tip post-dose at 30, 60, 90, 120 and 180 mins. Glucose levels were accessed using a blood glucose meter. Graphs show plasma glucose concentrations at the beginning, at week 6, week 24 and week 32 of APOE KO, APOEε3, APOEε4 and WT mice fed A, C, E, G) LFD and B, D, F, H) HFD. Values are mean±SEM, n = 10–12. *p<0.05, **p<0.01; ***p<0.001 versus WT mice by t-test. Significance is indicated by * APOE KO, ^+^ APOEε3, ∧ APOEε4.

Insulin resistance was induced by feeding APOE mice a high (60%) fat diet (HFD), or low (10%) fat diet (LFD) for 32 weeks. Throughout this period all APOE mice fed on a HFD gained more body weight in comparison to the LFD fed animals. Within the HFD group, APOE KO mice gained the most weight at the end of the 32 weeks (Figure 1). Oral glucose tolerance tests were taken at baseline and then at every 6 weeks following initiation of study. Prior to HFD feeding fasting plasma glucose levels were similar across genotypes (Figure 2A, B). At week 6 all HFD fed mice had developed impaired glucose tolerance. In LFD fed mice, plasma glucose levels reached the maximum at 30 min after glucose challenge; thereafter glucose elimination occurred by 60 min. In contrast, there was hardly any glucose elimination between 30 min and 60 min in HFD fed human APOEε3 and APOEε4 knock-in mice whilst in the WT and APOE KO mice glucose was eliminated at a much slower rate compared to LFD fed animals (Figure 2C, D). Over the duration of high fat feeding, glucose tolerance became increasingly impaired (Figure 2E-H).

**Figure 3:**
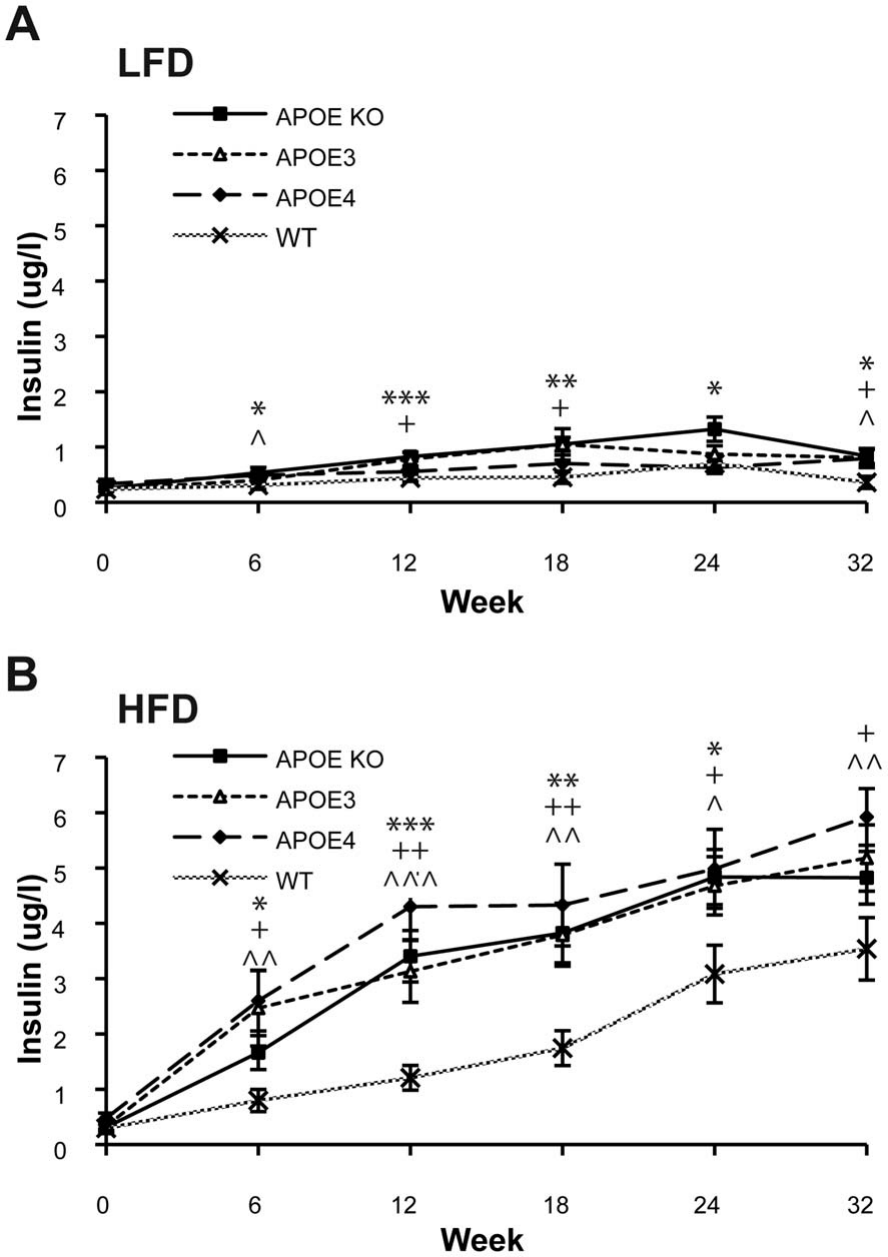
APOE mice develop diet-induced T2DM- like insulin resistance. Plasma insulin levels were measured in APOE KO, APOEε3, APOEε4 and WT mice over 32 weeks of feeding with A) LFD and B) HFD. Blood samples from the tail vein at each OGTT were collected and plasma was analysed for insulin by ELISA. Values are mean±SEM, n = 10–12. In A) plasma insulin levels in all LFD animals reached *p<0.05 by t-test compared to WT mice. In B) plasma insulin levels in HFD fed animals reached *p<0.05 or ***p<0.001 by t-test compared to WT mice as indicated. Significance is indicated by * APOE KO, ^+^ APOEε3, ∧ APOEε4.

Hyperglycaemia was accompanied by elevations in plasma insulin. At baseline all groups of mice had similar levels of insulin (Figure 3). At the end of the 32 weeks study, all the HFD group of animals had statistically significantly elevated levels of plasma insulin compared to the LFD group, with the highest in APOEε4 (5.9±0.5 µg/l), followed by APOEε3 (5.2±0.6 µg/), APOE KO (4.8±0.5 µg/l) and then WT (3.5±0.6 µg/l) (Figure 3B). Within each APOE genotype and between the LFD and HFD fed animals, insulin levels reached a significant difference by 6 weeks of diet. In contrast, insulin levels in the LFD fed animals increased by only a small amount (Figure 3A).

### Tau phosphorylation is reduced, but APP metabolism is unchanged, in HFD fed APOE mice

**Figure 4:**
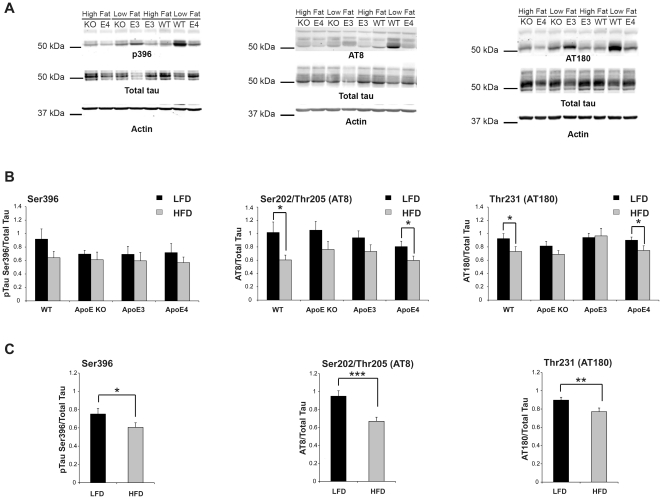
Tau phosphorylation is reduced in HFD fed APOE mice, independent of genotype. The frontal cortex from APOE mice were homogenised in sucrose homogenisation buffer, lysates were then immunoblotted with the indicated antibodies. A) Western blot of tau antibodies in frontal cortex of APOE KO, APOEε3, APOEε4 and WT mice fed LFD or HFD. B) Density of phosphorylated tau normalised against total tau. C) Tau phosphorylation is dependent on diet. Phosphorylated tau is normalised to total tau, data is grouped by diet. *p<0.05; **p<0.01; ***p<0.001 by t-test.

Comparing HFD to LFD animals we found a decrease in tau phosphorylation in the insulin resistant animals at all epitopes examined. This decrease was preserved across all genotypes with a significant decrease in tau phosphorylation in HFD fed WT animals at the Ser202/Thr205 (p = 0.010) and Thr231 epitopes (p = 0.042) and a trend towards reduction at Ser396 (Figure 4A and 4B). Analysis of all genotypes and all epitopes by ANOVA shows a significant interaction between diet and tau phosphorylation (p<0.001) but no effect of genotype. Combining genotypes shows a highly significant effect of tau phosphorylation at all three epitopes (Ser396 p = 0.03, Ser202/Thr205 (AT8) p = 0.0002 and Thr231 (AT180) p = 0.008) (Figure 4C). With respect to levels of soluble Aβ, we observed no significant changes in Aβ40 or Aβ42 according to diet within each APOE genotype nor did we observe changes in APP C-terminal fragments (APP-CTFs) (data not shown). Given the changes in tau phosphorylation we measured the level of active tau kinases and tau phosphatases; GSK-3αβ by using an activity dependent phospho-kinase antibody, Cdk5 by using p35/25 specific antibody and the tau-phosphatase, PP2A. We also measured the activity of Akt, a member of the insulin signalling pathway. No significant changes in the brain were found in GSK-3αβ, Cdk5, PP2A or Akt by APOE genotype or diet (data not shown).

### Pioglitazone treatment has genotypic specific effects on tau phosphorylation at the Ser202/Thr205 epitope

**Figure 5:**
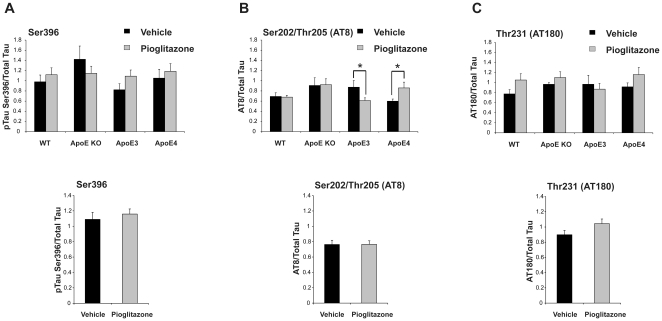
Pioglitazone treatment selectively lowers tau phosphorylation at the Ser202/Thr205 epitope. APOE mice fed HFD for 32 weeks were treated with pioglitazone or vehicle for the final 3 weeks. A) ptau Ser396, B) Ser202/Thr205 (AT8), C) Thr231 (AT180). *p<0.05 by t-test.

Previously, pioglitazone has been shown to alter insulin sensitivity in mice and has been suggested as a possible approach to disease modification in AD. Therefore we treated APOE mice with pioglitazone during the final three weeks of HFD feeding. Adiponectin was measured to show pioglitazone treatment was effective over time. Plasma adiponectin levels increased significantly after 3 weeks of drug treatment (data not shown) [37]. In WT animals there was no consistent and no significant effect on tau phosphorylation. However, there was a genotypic effect at the Ser202/Thr205 epitope with pioglitazone treated APOEε3 mice showing a decrease in tau phosphorylation (p = 0.012) and the reverse, a significant increase in tau phosphorylation in APOEε4 animals (p = 0.034) receiving pioglitazone treatment (Figure 5B). When combining genotypes we found no changes in tau phosphorylation based on pioglitazone or vehicle treatment groups in any of the three phospho-tau epitopes (Ser396, Ser202/Thr205 or Thr231) (Figure 5).

Given the change in tau phosphorylation at the Ser202/Thr205 epitope, again we measured the level of active tau kinases GSK-3αβ and Cdk5, the tau-phosphatase, PP2A and changes in Akt, a component of the insulin signalling pathway. However, we found no significant changes correlating to the genotypic effect (data not shown).

### Pioglitazone treatment on APP processing or soluble Aβ levels in APOE animals

**Figure 6:**
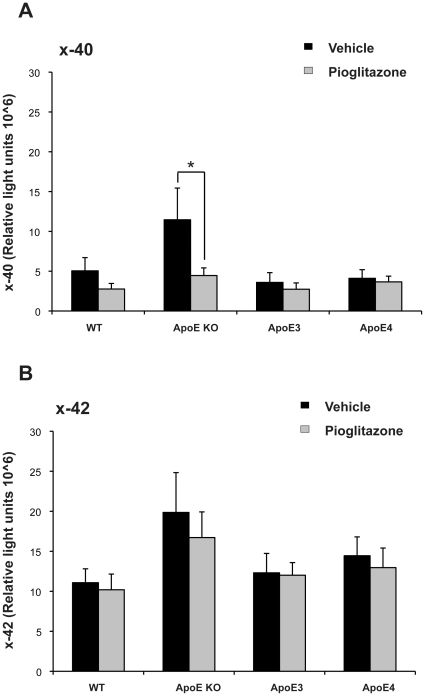
Levels of soluble Aβ40 and Aβ42 in HFD fed mice treated with or without pioglitazone. Aβ A) x-40 and B) x-42 was analysed by ELISA in the frontal cortex of APOE mice fed HFD and with or without pioglitazone treatment. *p<0.05 by t-test.

Pioglitazone treatment has been shown to lower amyloid load in animal models [33], [38]. In our study, APOE KO animals fed a HFD showed a trend for higher levels of soluble Aβ40 and Aβ42 compared to all other APOE genotypes (Figure 6). No effects of pioglitazone were observed in relation to Aβ42 at any genotype. No effects of pioglitazone on Aβ40 were observed in either WT or APOEε3 or APOEε4 animals but there was a significant reduction in APOE KO animals. We note however that APOE KO animals had a particularly high Aβ40 level pre-treatment and this may represent regression to the mean in a single genotype.

Changes in APP processing of holo APP to APP-CTFs (C83, C89 and C99) did not differ between treatment groups or within each APOE genotype (data not shown).

## Discussion

There is increasing awareness of modern lifestyles and their effect on disease progression; including the increasing prevalence of lifestyle induced T2DM. The risk of AD is increased in diabetics [9]. Although the mechanisms underlying this are not yet clear, evidence from animal models has provided some clues towards the underlying cause.

Previous *in vivo* studies investigating the effect of diabetes on AD pathology have produced ambiguous findings. The streptozotozin (STZ) approach, commonly used to model T1DM, has been shown to cause increased tau phosphorylation [39], [40], [41], [42], [43] and/or increased Aβ production in brain [41], [44], [45]. In the same way, T2DM models, for example db/db or high fat fed mice have shown increased levels of Aβ or increased tau phosphorylation and tau cleavage [43], [46]. However, other studies report no such changes [44], [47]. Further T2DM models combining diet with transgenic AD models have not only shown increased Aβ burden [18], [48] but also increased levels of tau [49]. Additionally, gene knockout models for IRS2 and the insulin receptor have shown increased tau phosphorylation [20], [21], [50] whilst other studies observed opposite findings [19].

Our results show a diet effect on tau phosphorylation; HFD reduces tau phosphorylation compared to LFD fed animals. This is contrary to a recent study finding no effect of diet on tau phosphorylation, despite an increase in tau gene expression [47]. As this was a study of young mice (4 weeks compared to 11 months in our study) and relatively short term diet (16 weeks compared to 32 weeks in our study) it is likely that effects on tau phosphorylation we observe are due to chronic HFD in older mice. Our observation also goes somewhat against the idea that peripheral hyperinsulinemia promotes tau pathology, as reported in an earlier study [19]. However, in this study an acute insulin challenge (20 min peripheral injection) was shown to increase tau phosphorylation. This is not comparable to the data reported here where tau phosphorylation is reduced in the context of chronic HFD induced hyperinsulinemia; arguably a model more reflective of diet induced hyperinsulinemia in man. In a recent clinical study by Reger and colleagues, intranasal insulin raised the level of brain insulin and improved memory in AD patients [25]. Improved cognitive performance has also been demonstrated in animals treated with intranasal insulin, which also improved diabetic brain neuropathy [27]. It is possible that the peripheral hyperinsulinemia in the HFD animals in this study may induce increased entry of insulin into the brain, which, *in the absence of brain insulin resistance*, results in a reduction in the levels of phosphorylated tau.

Furthermore in contrast to previous studies, we found tau phosphorylation was independent of APOE genotype [51], [52], [53], [54], [55]. Whilst we are unable to provide a full explanation for this, one possible source of discrepancy is the difference between our study and in both papers by Tesseur [52] and Harris [54] in the use of transgenic mice expressing APOEε3 and APOEε4 in neurons. Previously, cellular origin of apoE in the brain has been shown to effect its actions-no effect of genotype on apoE excitoprotection was observed when expressed in astrocytes whereas apoE4 but not apoE3 was excitotoxic when expressed in neurons [56]. It is possible that such differences in the models used in the experiments might underlie the differing observations.

The TZD, pioglitazone, is a potent synthetic PPARγ agonist that targets PPARs, members of the NRC1 class of the nuclear hormone receptor superfamily of ligand-activated transcription factors that are expressed in a variety of cell types. Pioglitazone has a higher permeability through the blood brain barrier (BBB) compared to rosiglitazone [57]. Our data shows that pioglitazone has an APOE genotype specific effect at the Ser202/Thr205 phospho-epitope of tau; in HFD fed and pioglitazone treated APOEε3 animals, a further reduction of tau phosphorylation was observed in comparison to ε3 animals treated with vehicle. The reverse, an increase in tau phosphorylation was observed in pioglitazone treated APOEε4 animals. Whilst in the APOE KO or murine APOE animals, no significant changes in tau phosphorylation by pioglitazone treatment were detected. Our results are consistent with results from a rosiglitazone clinical trial in which treatment improved cognition in APOEε4 negative patients with mild to moderate AD [30] and a recently published paper that observed APOE isoform dependent effects of rosiglitazone in adipose tissue [58]. As mentioned earlier, there is some evidence of a synergistic interaction between the APOEε4 allele and T2DM and the risk of AD is highest amongst APOEε4 carriers with T2DM [14]. Evidence on mitochondrial dysfunction as part of the pathophysiology of both AD [59] and T2DM [60] is also emerging. Greater mitochondrial dysfunction has been detected in AD APOEε4 carriers [61] and significant evidence show that this is the cause of apoE4 protein fragments [62], as apoE4 is more susceptible to degradation compared to the apoE2 and apoE3 isoforms [63]. One hypothesis is that the formation of C-terminal truncated fragments by neuron-specific cleavage of the apoE4 protein escapes through the secretory pathway, enters the cytosol and interacts with mitochondria via its hydrophobic lipid-binding region [64]. This leads to perturbation of mitochondrial function and hence diminishes mitochondrial participation in glycolytic processes. Via the same route apoE4 truncated fragments can also induce tau phosphorylation, as shown *in vivo* and *in vitro* [63], [64], [65], [66], [67], [68]. Since a direct role of PPARγ agonism is to stimulate mitochondrial biogenesis [69], [70], [71], [72], this suggests that perhaps neurotoxic C-terminal fragments in our APOEε4 mice caused excessive damage to mitochondria in neurons. Accordingly, APOEε4 mice did not respond to pioglitazone like APOEε3 mice. A higher dose of pioglitazone may possibly overcome the detrimental effects of apoE4 we observed.

According to the amyloid cascade hypothesis of AD, it is the formation or deposition of Aβ that initiates the formation of neurofibrillary tangles [73]. We examined the levels of soluble Aβ40 and Aβ42 by ELISA and also the processing of APP by western blotting in our insulin resistant animals with and without pioglitazone treatment. Surprisingly, we found no changes in APOE knock in animals. Our findings oppose the findings by two studies; Ho *et al*., who found diet-induced insulin resistance promoted generation of Aβ40 and Aβ42 in the brain of Tg2576 mice [18] and from a very recent study which found apoE isoform dependent effects on Aβ levels in PDAPP mice [74]. In both of these studies by Ho *et al*., and Bales *et al*., the use of APP transgenic animals predisposes the animals to amyloid pathology. This may allow both authors to detect an effect with diet or APOE genotype, which we failed to find. In our HFD fed APOE KO animals we detected a trend of higher levels of soluble Aβ40 and Aβ42 in comparison to animals with the APOE gene. The idea that APOE KOs cannot clear Aβ from brain has been suggested [75] and implies that the clearance of Aβ from the brain to the periphery requires apoE [74] and is mediated by transport of apoE-Aβ complexes across the BBB via LRP-1 [75]. Further, our results did not replicate the finding of reduction in Aβ levels following pioglitazone treatment, as reported in two earlier *in vivo* studies using Tg2576 mice [38] and APPV717l mice [33]. This is most likely due to different mouse models and/or diet may play a part in the formation of Aβ species. Numerous studies have reported the role of apoE in the periphery [76], in our study we report for the first time the combined effect of apoE and insulin in the brain *in vivo*. Our data therefore suggests that in our APOE model of diet-induced T2DM, tau phosphorylation arises due to some process downstream of Aβ.

In both studies; HFD versus LFD and HFD with pioglitazone treatment, we did not detect any changes in activation of members of the insulin signalling pathway (Akt, GSK-3αβ), tau kinases (GSK-3αβ, Cdk5) or tau phosphatase (PP2A). The lack of differences in Akt, GSK-3αv or Cdk5 phosphorylation indicates the possibility of secondary molecules affecting the reduction in tau phosphorylation. However, this was not due to PP2A as we detected no changes in this tau phosphatase. Despite our findings, many tau phosphatases and kinases have been identified to date [77] and further examination of these may help us identify the possible candidates responsible for dephosphorylation of HFD-induced tau phosphorylation, such as PKA, JNK, MAP Kinases and PTEN. Furthermore, it is probable that the period required to increase sufficient levels of insulin in the brains of these animals by HFD feeding lead to a failure in detecting changes in the insulin signalling cascade which may have occurred transiently at an earlier time point.

In conclusion, diet-induced insulin resistance appears to induce changes in tau phosphorylation due to peripheral hyperinsulinemia independent of APOE genotype. The lowering of tau phosphorylation by pioglitazone treatment in insulin resistant animals indicates that human APOEε3 is the isoform most responsive to treatment. However, prospective studies are required to elucidate the mechanism and to understand the relative importance between brain and peripheral insulin resistance.

## Methods

### Ethics Statement

All animal procedures were reviewed and approved by the GlaxoSmithKline Animal Care and Use Committee, and were performed in accredited facilities in accordance with institutional guidelines and the Guide for the Care and Use of Laboratory Animals (Institute of Laboratory Animal Resources, National Research Council). For the study, the Project License number was 80/01892 and the 3 SHOP numbers (protocols) were SP08-01474, SP08-03665 and SP09-00223. Both Project license number and SHOP went through the ethical review process.

### Animals and tissue preparation

Mice with human APOE genes replacing murine APOE genes (henceforth described as ‘APOE mice’) were generated as published [78], [79], [80]. In brief, the mice were created by gene targeting of the endogenous murine APOE gene locus with the human APOE alleles; APOEε3 or APOEε4. Consequently mice expressed human apoE isoforms under the control of murine APOE regulatory sequences and have very similar apoE expression levels to the non-transgenic apoE expressing mice.

#### Diet study

3 months old male APOE knockout, APOEε3-transgenic and APOEε4-transgenic mice, together with background strain C57BL6 mice were kept on a high fat (60% fat) (n = 12 per genotype) or low fat (10% fat) diet (n = 12 per genotype) (Research Diets, D12492 and D12450B respectively) for 32 weeks. Animals were housed individually under controlled conditions of temperature and lighting and given free access to food and water. Metabolic changes were assessed until insulin resistance developed in mice fed on the high fat diet (HFD).

#### Treatment study

All animals at 3 months of age were given a HFD (Research Diets, D12492) for 32 weeks. During the last 3 weeks of diet, treatment group animals (n = 12 per genotype) were orally dosed with pioglitazone (20 mg/kg p.o.) whilst the vehicle group (n = 12 per genotype) received 1% methylcellulose. Animals were dosed twice daily (8 am and 8 pm). On day 1 blood was removed by direct venopuncture from the tail vein before dosing began for baseline readings. An additional blood sample was collected following 3 weeks treatment and used to assess metabolic changes.

#### Assessment of metabolic changes

Glucose tolerance tests were conducted to assess the level of circulating glucose prior to commencing each study and then at 6 or 12 weekly intervals following the initiation of study. Animals were fasted overnight. The next morning, animals were placed in a warming chamber prior to blood sampling and blood was taken by direct venopuncture. Mice were then given a single oral dose of glucose (3 g/kg p.o dose volume 10 ml/kg) and serial blood samples collected from the tail tip post-dose at 30, 60, 90, 120 and 180 min. Glucose levels were accessed using a blood glucose meter (Ascensia, UK). Blood samples collected from the tail vein were centrifuged at 3000× g for 5 min, plasma was aliquoted into separate tubes then stored at -80°C until analysis with commercial ELISA kit for insulin (DRG, Germany).

#### Tissue preparation

At termination of each study brains were removed, the frontal cortex was subdissected and stored at -80°C until use.

### Protein extraction

The frontal cortex region was weighed whilst frozen and then homogenised rapidly in 10 fold volume of a detergent free tissue homogenisation buffer (250 mM sucrose, 20 mM Tris-HCl (pH 7.4), 1 mM EGTA, 1 mM EGTA, 100 mM PMSF, 1 mini complete protease inhibitor cocktail tablet (Roche, UK) and 1 mini complete phosphatase inhibitor cocktail tablet (Roche, UK)) on ice. A sample of total brain homogenate was collected and stored at −80°C until use in Aβ ELISA. Homogenates were then centrifuged at 47,000 rpm for 20 min at 4°C in a Beckman TLA 55 rotor (Beckman, Palo Alto, CA, USA). The soluble supernatant (S1 fraction) was collected and the pellet resuspended in a same volume of the above buffer with the addition of 1% Triton-X 100, then subjected to centrifugation again. The resultant supernatant (S2 fraction) was collected and stored at −80°C with the S1 fraction and the pellet, P2 until use.

### Western blotting

#### 8% Sodium dodecyl sulphate-polyacrylamide gel electrophoresis (SDS-PAGE)

S1 and S2 samples were diluted in an equal volume of 2× reducing laemmli sample buffer (BioRad, UK) and boiled at 100°C for 5 min. Samples were separated on 8% SDS-PAGE gels at 150 V for 1 h at room temperature (RT). Proteins were then transferred onto nitrocellulose membranes at 80 V for 1 h at RT. Blots were incubated in a blocking solution containing 5% skimmed milk (Marvel) in phosphate buffered saline with 0.1% tween (PBST) for 1 h at RT then probed with primary antibody overnight at 4°C. The blots were washed three times with PBST then incubated with either goat anti-rabbit or goat anti-mouse secondary antibodies conjugated to fluorophors (Molecular Probes, Invitrogen) emitting at wavelengths of either 700 or 800 nm for 1 h at RT. Blots were washed three times again with PBST. Proteins were detected using an Odyssey infrared imager (Licor, UK) and densitometry performed using ImageJ.

#### 16% Tricine gradient gels

S2 samples were diluted in an equal volume of 2× Tris-glycine SDS sample buffer containing NuPage sample reducing agent (Invitrogen, UK) and heated for 3 min at 85°C. Samples were spun briefly then separated on 16% Tricine gradient gels (Invitrogen, UK) at 130 V for 2 h at RT. Proteins were then transferred onto 0.2 µm pore size nitrocellulose membranes (Invitrogen, UK) at 200 mA for 2 h at RT. Blots were boiled by microwave for 5 min in PBS then incubated in a blocking solution for 1 h at RT then probed with primary antibody overnight at 4°C. The blots were washed three times with PBST then incubated with either goat anti-rabbit or goat anti-mouse horse radish peroxidase (HRP)-conjugated secondary antibodies (GE Healthcare, UK) for 1 h at RT. Blots were washed three times again with PBST. Immunoreactivity was visualised using enhanced chemiluminescence reagents (GE Healthcare, UK), x-ray film (Fuji, UK) and developer. ImageJ was used for quantification.

### Antibodies

All antibodies (table 1) were used at 1∶1000 dilution in blocking solution PBST containing 5% milk. Protein bands were normalised to β-actin antibody purchased from Abcam, used at 1∶5000 dilution. Fluorophore tagged secondary antibodies (Molecular Probes, Invitrogen) were all used at 1∶6000. All HRP-conjugated secondary antibodies (GE Healthcare, UK) were used at 1∶5000.

**Table 1:** List of primary antibodies.

Antibody	Type	Source
AT8 (Ser 202/Thr205)	Mouse monoclonal	Pierce
AT180 (Thr231)	Mouse monoclonal	Pierce
Phospho Tau (Ser396)	Mouse monoclonal	Cell Signaling Technology
Total Tau	Rabbit polyclonal	Cell Signaling Technology
Phospho-GSK-3αβ (Ser21/9)	Rabbit polyclonal	Cell Signaling Technology
Total GSK-3αβ	Mouse monoclonal	Stressgen
Phospho-Akt (Ser473)	Rabbit polyclonal	Cell Signaling Technology
Total Akt	Rabbit polyclonal	Cell Signaling Technology
Phospho-PP2A (Tyr307)	Rabbit polyclonal	Cell Signaling Technology
Total PP2A	Goat polyclonal	Santa Cruz
p35/25	Rabbit monoclonal	Cell Signaling Technology
Anti-APP 369	Rabbit polyclonal	J Buxbaum

### Abeta ELISA

Levels of mouse soluble Aβ 1-40 and Aβ 1-42 were measured using the chemiluminescent BetaMark Beta Amyloid x-40 and x-42 ELISA kits (Covance) according to the manufacturer's instructions. Briefly, total brain homogenates from the frontal cortex were diluted in working incubation buffer containing HRP detection antibody then loaded in duplicate onto the antibody coated plate. The ELISA plate was incubated overnight at 4°C. The plate was washed 5 times before addition of chemiluminescent substrates and read using a Wallac luminometer (Perkin Elmer).

### Statistics

Statistical significance was performed using the student's t-test and p<0.05 was considered significant. Data are presented as mean±SEM.
